# Cerebrovascular Diagnoses During First Recorded Pregnancies in a 17-Year Period—A Nationwide Analysis of Healthcare Administrative Records Between 2004 and 2020 in a Central-Eastern European Population

**DOI:** 10.3390/epidemiologia7030080

**Published:** 2026-06-05

**Authors:** Dániel Bereczki, Péter Vinnai, Mónika Bálint, Ferenc Oberfrank, Balázs Dobi, Dániel Bereczki, Ildikó Vastagh

**Affiliations:** 1 János Szentágothai Doctoral School of Neurosciences, Semmelweis University, 1085 Budapest, Hungary; bereczki.daniel@hotmail.com; 2 Department of Neurology, Bajcsy-Zsilinszky Hospital and Clinic, 1106 Budapest, Hungary; vastagh.ildiko@bajcsy.hu; 3 HUN-REN–SU Neuroepidemiological Research Group, Semmelweis University, 1083 Budapest, Hungary; vinnai.p@gmail.com (P.V.); balint.moni@gmail.com (M.B.); balazs.dobi@gmail.com (B.D.); 4 Hungarian Academy of Sciences, 1051 Budapest, Hungary; oberfrank.ferenc@titkarsag.mta.hu; 5 Department of Neurology, Semmelweis University, 1083 Budapest, Hungary

**Keywords:** epidemiology, big data, women, cerebrovascular diagnoses, Hungary, pregnancy

## Abstract

Introduction: Cerebrovascular disorders are major contributors to maternal morbidity and mortality during pregnancy. In this nationwide study in Hungary, we evaluated the frequency of cerebrovascular diagnoses during first recorded pregnancies in a 17-year period. Material and Methods: In the framework of the NEUROHUN project utilising nationwide administrative healthcare data, we included women with at least one delivery and with at least one cerebrovascular diagnosis during their first pregnancies recorded between 2004 and 2020. To minimise the number of misclassified first pregnancies due to database limitations appearing towards the beginning of the database, trend analyses using linear regression models were restricted to the 2011–2020 period. Results: During first recorded pregnancies in the 17-year study time frame (*n* = 952,451), the frequency of ICD-10 cerebrovascular diagnoses was 0.17% (*n* = 1614), with an estimated overall prevalence rate of 169.4 per 100,000 women (95% CI: 161.4–177.9). Transient ischaemic attack (TIA) was the most prevalent specific diagnosis, with a rate of 72.7 per 100,000 (95% CI: 67.4–78.3). In a multiple linear regression model on the mean age at first recorded birth within 2004–2020, women diagnosed with a cerebrovascular disorder were, on average, 1.935 years older at the time of their first birth compared to those without a diagnosis (mean difference: 1.935 years; 95% CI [1.188–2.682], *p* < 0.001). This analysis, adjusted for calendar year trends between 2011 and 2020, suggests that higher maternal age is an important factor associated with these events. In a sensitivity analysis of the linear regression using the diagnoses of G45, I60, I61, I63, and I67 we found that the relationship between the presence of diagnosis and mean age remained significant in the case of G45, I63 and I67, but not for I60 and I61. In the logistic regression model, compared to the reference group of women < 25 years, the prevalence for all evaluable cerebrovascular diagnoses was significantly higher in the >34 age group, and was also significantly higher for TIA (G45) and cerebral infarction (I63) diagnoses in the 25–34 age group. The rate of cerebral infarction among cerebrovascular disorders showed an increasing trend towards higher maternal age (<25 years age group: 12%; 25–34 years age group: 16.5%; >34 years age group: 20.0%). Also, when compared to the reference category of diagnosed women < 25 years, the increase in the odds of cerebral infarction was significant at the 5% level among women > 34 years. In contrast, there was no increment in the proportion of intracranial bleedings at older age. Discussion and Conclusion: The prevalence of most cerebrovascular diagnoses increases significantly with higher maternal age. Allowing for the limitations of our study, we found that in a Central-Eastern European population, the prevalence of cerebrovascular diagnoses during first recorded pregnancies between 2004 and 2020 was 169.4 per 100,000 (0.17%), with TIA being the most common diagnosis in approximately one-third of cases. The rate of cerebral infarctions among cerebrovascular diagnoses was almost twice as high in those over 34 years of age compared to those below 25. The frequency of pregnancy-related ischemic strokes and cerebral haemorrhages in the Central-Eastern European population corresponds to published values.

## 1. Introduction

Neurological disorders of vascular origin are major contributors to maternal morbidity and mortality during pregnancy. Pregnant women are at risk of all stroke subtypes, i.e., ischaemic stroke, haemorrhagic stroke and cerebral venous thrombosis [1]. During pregnancy, the pathomechanisms of ischaemic strokes include mainly cardioembolism, paradoxical embolism through a patent foramen ovale, cervical artery dissection and severe vasospasm, while haemorrhagic strokes can be a result of intracerebral haemorrhage or subarachnoid haemorrhage [2]. It is to be noted, that pregnancy and puerperium are important risk factors for cerebral venous thrombosis resulting in venous infarction, with or without haemorrhagic conversion [3]. In general, stroke affects 30 per 100,000 gestations and thereby pregnancy exposes a 3-fold risk compared to the general young adult population [4]. It should also be taken into account, however, that there is a considerable variability in reported prevalence with numbers ranging from 4.3 to 210 per 100,000 deliveries [5]. The higher stroke risk during pregnancy can be attributed to several factors. First, there are physiological changes in haemodynamics and haemostasis including vasodilation, venous congestion, hypervolemia, increased cardiac output, higher level of prothrombotic factors (e.g., factors VII, IX, X, XII and XIII, fibrinogen, von Willebrand factor) and a decreased activity of antithrombotic molecules (e.g., reduced protein S activity, acquired protein C resistance). Second, there are pathological states specific to pregnancy resulting in a higher risk of stroke, e.g., gestational hypertension, preeclampsia/eclampsia, gestational diabetes, peripartum cardiomyopathy or amnion fluid embolism [6]. Cerebrovascular accidents are responsible for 7.6% of pregnancy-related deaths in the United States [7] and in women younger than 35 years, 18% of strokes are associated with pregnancy [8]. Notably, the prevalence of pregnancy-related neurological diseases of vascular origin seems to rise worldwide [9,10,11]. Some of the main reasons for this include increasing maternal age, the rising rates of cerebrovascular risk factors such as obesity, hypertensive disorders, diabetes, or heart disease and advances in the medical care of certain chronic diseases [9]. The impact of cerebrovascular events during pregnancy justifies the need for comprehensive epidemiological research. From Europe, population-based data of this patient group are scarce. While there are such studies from France [12], England [13], Finland [14] and Sweden [15], publications from Central or Eastern European countries are lacking. The less pronounced improvement in the incidence of ischemic stroke between 1991 and 2021 in Central and Eastern Europe compared to Western European Countries [16] underlines the need for such data from this European region.

In this nationwide study in Hungary, our aim was to evaluate the frequency of maternal cerebrovascular diagnoses during first recorded pregnancies in a 17-year period by utilising the NEUROHUN database [17].

## 2. Material and Methods

The NEUROHUN 2004–2020 project was created within the framework of the Hungarian National Brain Research Program by applying healthcare administrative reports submitted for reimbursement purposes to the National Healthcare Fund from all hospitals and specialist outpatient services throughout Hungary. As the NEUROHUN database has already been proven useful in characterising epidemiological features of various neurological conditions [18,19,20,21,22,23], this collection of data provided the basis and has been further analysed in our retrospective study. Approval was provided by the Ethics Committee of Semmelweis University, Budapest, Hungary (Approval No.: SE TUKEB 88-1/2015) and by the Medical Research Council (ETT IV/7136-1/2021/EKU).

In the current study, women with at least one delivery during 2004–2020 and with at least one cerebrovascular diagnosis during their first recorded pregnancies within this time period were included. Reports submitted by general practitioners and by non-clinical specialty areas (e.g., laboratory diagnostics, diagnostic imaging, physiotherapy, psychology, etc.) have not been analysed, thus only diagnoses confirmed by any clinical specialties (i.e., either neurologists or non-neurologists) were covered. Cerebrovascular disorders were identified by three-digit codes (G45, G46, I60–I69) from the 10th International Classification of Diseases (ICD-10) [24] and consisted of diagnostic groups detailed in Table 1. Specific ICD-10 codes (O60: Preterm labour and delivery, O80: Single spontaneous delivery, O81: Single delivery by forceps and vacuum extractor, O82: Single delivery by caesarean section, O83: Other assisted single delivery, O84: Multiple delivery) were used to record the type of delivery. The dates of the labour and of the cerebrovascular diagnoses enabled the identification of those patients who received a certain cerebrovascular ICD-10 code within nine months before their first recorded deliveries. During the study, we focused on pregnancies which ended with livebirth. Hence, gestations which terminated in miscarriage or stillbirth, were not included in the present evaluation.

## 3. Statistical Methods

Data management was in line with personal data protection regulations. During data anonymization, encrypted codes derived from original patient identifiers were used, thereby making record linkage possible. If a subject received the same diagnosis multiple times, the first appearance was taken into account.

Primary data extraction was performed by an IT specialist research assistant, with extensive experience in analysing medical records of patients with neurological diseases. Results of the database screening were transferred to Microsoft Excel and the R programming language (version 4.1.3) for further evaluation during the final analysis.

**Figure 1:**
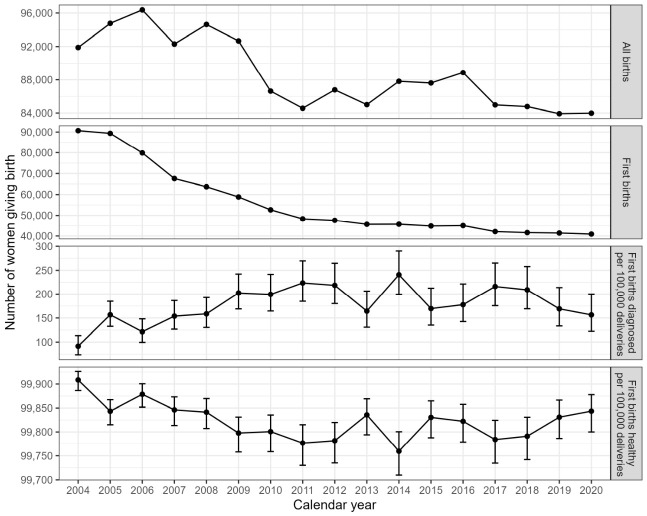
Upper panels: number of women giving birth in the complete study period of 2004–2020 (all births, first births). Lower panels: prevalence of first births with a cerebrovascular diagnosis, and first births without any cerebrovascular diagnoses recorded during pregnancy (presenting 95% confidence intervals).

The basic descriptive analysis on frequency data was derived from the total study period of 2004–2020. Trend analyses using linear regression models were restricted to 2011–2020 in order to minimise the effect of misclassified first pregnancies due to database limitations appearing towards the beginning of the database (Figure 1).

Prevalence rates for each cerebrovascular diagnostic category were calculated as the number of affected women divided by the total number of women with at least one delivery during the study period, expressed per 100,000 deliveries.

Multiple linear regression analysis was employed to estimate the mean difference in maternal age between women with and without cerebrovascular diagnoses. The model included the calendar year as a continuous covariate to account for potential temporal shifts in childbearing age. Estimates are reported as regression coefficients representing the mean age difference in years, alongside 95% confidence intervals and *p*-values.

Linear regression analysis was used to (1) assess the effects of calendar year (i.e., time-trend) and presence of cerebrovascular diagnosis on the mean maternal age at first recorded birth, and (2) to assess the effect of the maternal age on the ratio of diagnosed women at first recorded birth within the studied years.

The relative proportion of specific diagnoses among all cerebrovascular disorders and among all first births per age group was assessed using logistic regression analysis. Only diagnoses with at least 10 patients in the reference category of <25 years old patients were tested.

95% confidence intervals for ratios were calculated using the Agresti–Coull method, and for mean ages per calendar year using normal approximation.

A sensitivity analysis was performed using the diagnoses of G45, I60, I61, I63, and I67 separately to check if the associations seen for the overall diagnosed population persisted within the most relevant/prevalent diagnoses.

## 4. Results

**The overall frequency of cerebrovascular disorders among pregnant women**

By the use of our inclusion criteria, 952,451 women have been identified with at least one delivery during 2004–2020. Within the studied time frame 1614 women received any kind of cerebrovascular diagnosis during their first recorded pregnancy. Hence, 0.17% of all women with at least one labour during the 17-year period received at least one neurological diagnosis of vascular origin during their first recorded pregnancies between 2004 and 2020.

The frequency of individual cerebrovascular diagnoses is detailed in Table 1. By far the most frequent diagnosis was “Transient cerebral ischaemic attacks and related syndromes” (G45) affecting 692 women with a frequency of 72.7 per 100,000 deliveries. The second category affecting 376 individuals with a frequency of 39.5 per 100,000 was “Other cerebrovascular disorders” (I67). With similar numbers, “Cerebral infarction” (I63) ranked third with 319 patients affecting 33.5 per 100,000 deliveries.

**Diagnostic categories and maternal age at first recorded delivery during the study**

Among all 952,451 women with at least one delivery during the complete study period (2004–2020), the mean age at first recorded labour within the 17-year period was 29.0 years. Women with any cerebrovascular diagnosis recorded in the 9 months before delivery in the full study time frame (2004–2020) had their first recorded labour at the age of 31.2 years. The mean age was even higher with 31.5 years in the I60–I69 diagnostic subgroup (Table 1). In the total study period of 2004–2020, the oldest mean maternal age was found among women with “Sequelae of cerebrovascular disease” (I69) with 32.3 years at first recorded delivery. Further diagnostic groups are detailed in Table 1.

To minimise the effect of misclassified first pregnancies due to database limitations in the initial years of the database, we have performed a trend analysis for first recorded pregnancies only for the years of 2011–2020. Women in this analysis had no delivery in the 2004–2010 period. In this evaluation we investigated the effect of the presence of any cerebrovascular diagnosis, of the calendar year, and their interaction on maternal age with trend analysis in a multiple linear regression model.

**Figure 2:**
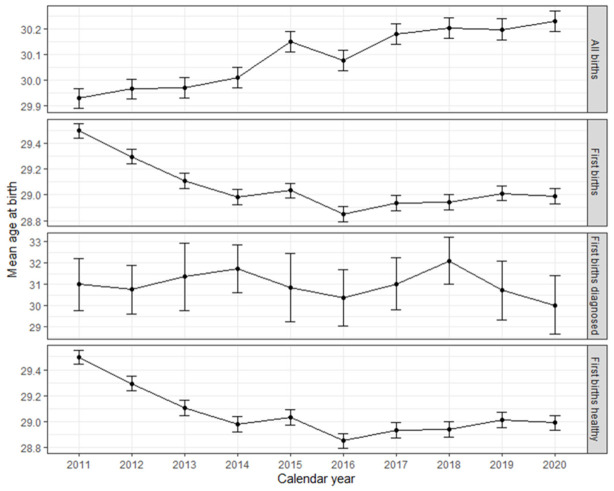
Mean age at birth with 95% confidence intervals in the restricted period of 2011–2020 (all births, first births, first births with a cerebrovascular diagnosis, and first births without any cerebrovascular diagnosis recorded during pregnancy).

We found that women with any cerebrovascular diagnosis were significantly older on average by 1.935 years (CI95% [1.188–2.682], *p* < 0.001) compared to women without diagnoses, while calendar years in the 2011–2020 period were not associated with the mean age at first labour (*p* = 0.351). The relationship between the variables is visualised in Figure 2. A sensitivity analysis of the linear regression was performed using the diagnoses of G45, I60, I61, I63, and I67, separately. It was found that the relationship between the presence of diagnosis and mean age remained significant in the case of G45, I63 and I67, but not for I60 and I61. In addition, the linear effect of the calendar year was also significant for G45, with an average increase in age of 0.200 per calendar year (CI95% [0.025–0.375], *p* = 0.039).

**Age groups and cerebrovascular diagnoses**

Of the 952,451 women 206,216 were below 25 years of age, 576,237 were between 25 and 34 years, and 163,661 were >34. No age data were available for 6337 women (<0.7%). Table 2 presents the distribution of each cerebrovascular diagnosis within three different age groups (<25 years; 25–34 years; >34 years) at first recorded delivery during the full time frame of the study (2004–2020).

In Table 3 the prevalence of cerebrovascular diagnoses within first births per age category (<25, 25–34, >34 years) per 100,000 deliveries is presented.

The relative proportion of “Cerebral infarction” (I63) among all cerebrovascular disorders nearly doubled with advancing maternal age, rising from 12.0% in women under 25 to 20.0% in those over 34. While “Transient cerebral ischaemic attacks and related disorders” (G45) remained the most common diagnosis across all cohorts (33.6–38.5%), higher maternal age was associated with a significant shift toward cerebral infarction, whereas the proportion of intracranial haemorrhages remained stable across age groups. Hence, higher maternal age was associated with a higher proportion of cerebral infarction compared to intracranial bleedings.

Regarding the category of “Cerebral infarction” (I63), when compared to the reference category of diagnosed women < 25 years, the increase in the odds of this diagnosis was significant at 10% significance level among women between 25 and 34 years and was significant at the 5% level among women > 34 years (Table 4).

When investigated within the number of all first births within a given age category, the increase in the odds of the diagnoses was significant among women between 25 and 34 years for G45 and I63 when compared to the reference category of diagnosed women < 25. For women > 34 vs. <25 years, all 6 diagnoses with at least 10 patients in the reference category showed significant increase in the odds at the 5% level (Table 5).

## 5. Discussion

In the present nationwide analysis of healthcare administrative records, our aim was to assess cerebrovascular diagnoses appearing during first recorded pregnancies in a 17-year period. This is the first large population-based report from the Central and Eastern European region that evaluates pregnancy-related cerebrovascular events in close to a million births over a 17-year period in the total population of a country.

We found that despite the young age of this population, the frequency of cerebrovascular diagnoses during a first recorded pregnancy between 2004 and 2020 was considerably high. As part of the study, we investigated the relationship between maternal age and the frequency of cerebrovascular diagnoses. In the population of patients with cerebrovascular diagnoses, we found that these women had a significantly higher maternal age on average.

With trend analyses (2011–2020), we found that women with any cerebrovascular diagnoses were significantly older on average compared to women with no such diagnoses while calendar years were not associated with the mean age at first recorded labour within this time frame.

With higher maternal age, the proportion of cerebral infarction diagnoses among all cerebrovascular diseases rose significantly, whereas the rate of intracerebral bleeding diagnoses did not increase. When the proportion of the diagnoses were investigated within the number of all first births per age category, the increase in the odds of the diagnoses was significant among women between 25 and 34 years for G45 and I63 when compared to the reference category of diagnosed women < 25. For women > 34 vs. <25 years, all 6 evaluable diagnoses with at least 10 patients in the reference category showed significant increase in the odds.

As a first step, we determined the frequency of all cerebrovascular diagnoses, which appeared in 0.17% among all first recorded pregnancies during the 17 years of the study. Study design, sample size, ethnic origin and mainly, the definition used for cerebrovascular diseases applied in different studies can result in considerable discrepancies regarding disease prevalence [25].

The relatively high frequency in our study can be attributed to the inclusion of all cerebrovascular diagnoses, including transient ischemic attacks, sequelae of cerebrovascular disease, and other cerebrovascular diseases in addition to frank strokes, i.e., cerebral infarction and intracranial bleedings. Furthermore, temporary symptoms might have been misinterpreted as cerebrovascular disorders. For these reasons, we performed a sensitivity analysis for the subgroups of cerebral infarctions (I63) and intracranial haemorrhages (I60 and I61). According to a systematic review [4], the prevalence of pregnancy associated non-haemorrhagic stroke was around 19.9/100,000, and for haemorrhagic stroke the value was 12.2/100,000, similar to our values of 33.5/100,000 for cerebral infarctions (I63) and 14.9/100,000 for haemorrhagic strokes (I60, I61).

With growing maternal age, the ratio between ischemic and haemorrhagic strokes shifted towards the ischemic subtype. This tendency was in line with literature data showing a higher proportion of haemorrhagic strokes among stroke subtypes in young patients [26].

According to our study, diagnosis of transient ischemic attacks had a frequency of 72.7 per 100,000 deliveries. In the case of transient neurological symptoms in young women, alternative diagnoses, especially migraine auras with no migraine history or migraine auras without headache, should also be considered [27]. Due to diagnostic difficulties, some transient symptoms (e.g., dizziness, fainting, numbness) may have been misclassified as those of vascular origin as well.

“Other cerebrovascular disorders” (I67) being the second most common diagnosis includes several conditions, e.g., dissection of cerebral arteries, non-ruptured cerebral aneurysm, cerebral atherosclerosis, hypertensive encephalopathy, cerebral venous thrombosis or cerebral arteritis, some of which are relatively common conditions in this specific population. For example, gestation is a well-known risk factor for cerebral venous thrombosis which accounts for 6–64% of pregnancy-related strokes [28]. As this group consists of various different medical entities, with very different mechanisms and relevance to pregnancy, overinterpretation of this category being the second most frequent should be carefully considered.

In the 2010s, the mean maternal age in most OECD (Organization for Economic Co-operation and Development) countries has risen to >30 years [29,30]. The trend toward delayed childbearing results in the increasing prevalence of hypertensive disorders and cardiac diseases among pregnant women and thus, in a growing number of pregnancy-related strokes [25,31].

Major strengths of our study were the analysis of reports from a single-payer state health insurance system enabling full coverage of national data, combined with a long study time period of 17 years (2004–2020) resulting in close to one million first recorded deliveries.

Also, our study had numerous limitations:

- Although the study period was long, it should be taken into account that first recorded pregnancies were not really first pregnancies in some of the cases. Restricting the trend analyses to the 2011–2020 period implies that women in these analyses definitely had no pregnancies in the 2004–2010 period of 7 years.
- The study involved subjects with livebirths, hence women with abortions or stillbirths were not included in the study.
- Although cerebrovascular diagnoses were associated with higher maternal ages, there was no adjustment for other risk factors (e.g., hypertension, diabetes, thrombophilia, etc.), hence no adequate support on causal or independent associations could have been made.
- The lack of primary care data and the inclusion of diagnosis given by any clinical specialties should be taken into account when evaluating completeness of the database and diagnostic specificity.
- The anonymized healthcare administrative database used in our study did not allow to identify individual patients; therefore, it was not possible to validate individual diagnoses.

## 6. Conclusions

The study reports frequencies of administrative cerebrovascular diagnoses in first recorded pregnancies, rather than validated incident cerebrovascular events during true first pregnancies. Our data confirm that the frequency of pregnancy-related cerebrovascular disorders, and specifically ischemic strokes and cerebral haemorrhages in the Central-Eastern European region is in the range of published values.

## Figures and Tables

**Table 1:** Classification of patients by cerebrovascular diagnosis received during first recorded pregnancy in the complete study period with prevalence and 95% CI and mean maternal age at first recorded delivery during 2004–2020.

Diagnostic Category (ICD-10 Code)	No. of Women *	Prevalence and 95% CI (Per 100,000 Deliveries)	Mean Maternal Age at First Recorded Delivery
All women with at least one delivery	952,451		29.0
All cerebrovascular disorders (I60–I69 + G45–G46)	1614	169.4 (161.4–177.9)	31.2
I60-I69 (ICD-10 group of “Cerebrovascular diseases”)	996	104.6 (98.3–111.3)	31.5
Transient cerebral ischaemic attacks and related syndromes (G45)	692	72.7 (67.4–78.3)	31.0
Other cerebrovascular diseases (I67)	376	39.5 (35.7–43.7)	31.2
Cerebral infarction (I63)	319	33.5 (30.0–37.4)	32.1
Sequelae of cerebrovascular disease (I69)	113	11.9 (9.9–14.3)	32.3
Occlusion and stenosis of precerebral arteries, not resulting in cerebral infarction (I65)	107	11.2 (9.3–13.6)	31.2
Subarachnoid haemorrhage (I60)	96	10.1 (8.2–12.3)	32.1
Intracerebral haemorrhage (I61)	46	4.8 (3.6–6.5)	30.7
Occlusion and stenosis of cerebral arteries, not resulting in cerebral infarction (I66)	38	4 (2.9–5.5)	30.5
Stroke, not specified as haemorrhage or infarction (I64)	36	3.8 (2.7–5.2)	31.5
Vascular syndromes of brain in cerebrovascular diseases (G46)	32	3.4 (2.4–4.8)	29.6
Cerebrovascular disorders in disease classified elsewhere (I68)	13	1.4 (0.8–2.4)	29.8
Other nontraumatic intracranial haemorrhage (I62)	8	0.8 (0.4–1.7)	30.8

* It is to be noted that an individual woman can appear in multiple different single ICD-10 diagnostic categories.

**Table 2:** Number of women classified in specific age groups at first delivery receiving cerebrovascular diagnoses during first recorded pregnancy in the complete study period of 2004–2020 (percentage of all cerebrovascular diagnoses within age group indicated in brackets).

Diagnostic Category (ICD-10 Code)	No. of Women (All Age Groups)	No. of Women (<25 Years)	No. of Women (25–34 Years)	No. of Women (>34 Years)
Transient cerebral ischaemic attacks and related syndromes (G45)	692 (36.9%)	91 (37.8%)	408 (38.5%)	193 (33.6%)
Other cerebrovascular diseases (I67)	376 (20.0%)	55 (22.8%)	205 (19.3%)	116 (20.2%)
Cerebral infarction (I63)	319 (17.0%)	29 (12.0%)	**175 (16.5%)**	***115 (20.0%)***
Sequelae of cerebrovascular disease (I69)	113 (6.0%)	12 (5.0%)	56 (5.3%)	45 (7.8%)
Occlusion and stenosis of precerebral arteries, not resulting in cerebral infarction (I65)	107 (5.7%)	16 (6.6%)	62 (5.8%)	29 (5.0%)
Subarachnoid haemorrhage (I60)	96 (5.1%)	11 (4.6%)	49 (4.6%)	36 (6.3%)
Intracerebral haemorrhage (I61)	46 (2.5%)	6 (2.5%)	32 (3.0%)	8 (1.4%)
Occlusion and stenosis of cerebral arteries, not resulting in cerebral infarction (I66)	38 (2.0%)	6 (2.5%)	23 (2.2%)	9 (1.6%)
Stroke, not specified as haemorrhage or infarction (I64)	36 (1.9%)	5 (2.1%)	19 (1.8%)	12 (2.1%)
Vascular syndromes of brain in cerebrovascular diseases (G46)	32 (1.7%)	8 (3.3%)	16 (1.5%)	8 (1.4%)
Cerebrovascular disorders in disease classified elsewhere (I68)	13 (0.7%)	1 (0.4%)	10 (0.9%)	2 (0.3%)
Other nontraumatic intracranial haemorrhage (I62)	8 (0.4%)	1 (0.4%)	5 (0.5%)	2 (0.3%)
**Sum of diagnoses**	1876	241	1060	575
**I63/(I60 + I61 + I62) diagnosis ratio**	319/150 (68.0%/32.0%)	29/18 (61.7%/38.3%)	175/86 (67%/33%)	115/46 (71.4%/28.6%)

Bold values highlight categories that had a significantly different ratio of diagnosis compared to the <25 years old reference category at 10% significance level and bold values in italics highlight ones significant at the 5% level; only diagnoses with at least 10 patients in the reference category were tested (values are detailed in Table 4).

**Table 3:** Prevalence of cerebrovascular diagnoses within first births per age category (<25, 25–34, >34 years) per 100,000 deliveries with 95% confidence intervals *.

Diagnostic Category (ICD-10 Code)	Prevalence in Women (<25 Years)	Prevalence in Women (25–34 Years)	Prevalence in Women (>34 Years)
Transient cerebral ischaemic attacks and related syndromes (G45)	44.1 (35.9–54.2)	***70.8 (64.3–78)***	***117.9 (102.4–135.8)***
Other cerebrovascular diseases (I67)	26.7 (20.4–34.8)	**35.6 (31–40.8)**	***70.9 (59.1–85)***
Cerebral infarction (I63)	14.1 (9.7–20.3)	***30.4 (26.2–35.2)***	***70.3 (58.5–84.4)***
Sequelae of cerebrovascular disease (I69)	5.8 (3.2–10.3)	9.7 (7.5–12.6)	***27.5 (20.5–36.9)***
Occlusion and stenosis of precerebral arteries, not resulting in cerebral infarction (I65)	7.8 (4.7–12.7)	10.8 (8.4–13.8)	***17.7 (12.2–25.6)***
Subarachnoid haemorrhage (I60)	5.3 (2.8–9.7)	8.5 (6.4–11.3)	***22 (15.8–30.5)***
Intracerebral haemorrhage (I61)	2.9 (1.2–6.5)	5.6 (3.9–7.9)	4.9 (2.3–9.8)
Occlusion and stenosis of cerebral arteries, not resulting in cerebral infarction (I66)	2.9 (1.2–6.5)	4 (2.6–6)	5.5 (2.7–10.6)
Stroke, not specified as haemorrhage or infarction (I64)	2.4 (0.9–5.9)	3.3 (2.1–5.2)	7.3 (4–13)
Vascular syndromes of brain in cerebrovascular diseases (G46)	3.9 (1.8–7.8)	2.8 (1.7–4.5)	4.9 (2.3–9.8)
Cerebrovascular disorders in disease classified elsewhere (I68)	0.5 (0–3)	1.7 (0.9–3.2)	1.2 (0–4.8)
Other nontraumatic intracranial haemorrhage (I62)	0.5 (0–3)	0.9 (0.3–2.1)	1.2 (0–4.8)
All first births with known age	206,216	576,237	163,661

* Confidence interval values under 0 were corrected to display 0. Bold values highlight categories that had a significantly different ratio of diagnosis compared to the <25 years old reference category at 10% significance level and bold values in italics highlight ones significant at the 5% level; only diagnoses with at least 10 patients in the reference category were tested (*p* values are detailed in Table 5).

**Table 4:** The *p* values for logistic regression analyses of data in Table 2 when compared to all cerebrovascular disorders *. Comparison of the odds of diagnosis in age groups with <25 years as the reference group.

ICD-10 Category	Age Groups		
	<25 Years	25–34 Years	>34 Years
**G45**	Reference	0.8284	0.4429
**I60**		0.9697	0.2804
**I63**		**0.082** **0**	***0.0029***
**I65**		0.6387	0.4410
**I67**		0.2133	0.5711
**I69**		0.8487	0.1116

* Only selected ICD-10 categories were tested due sample size limitations. Bold values highlight categories that had a significantly different ratio of diagnosis compared to the <25 years old reference category at 10% significance level and bold values in italics highlight ones significant at the 5% level; only diagnoses with at least 10 patients in the reference category were tested.

**Table 5:** The *p* values for logistic regression analyses of data in Table 3 when compared to all first births *. Comparison of the odds of diagnosis in age groups with <25 years as the reference group.

ICD-10 Category	Age Groups		
	<25 Years	25–34 Years	>34 Years
**G45**	Reference	***<0.0001***	***<0.0001***
**I60**		0.1622	***<0.0001***
**I63**		***0.0001***	***<0.0001***
**I65**		0.2436	***0.0080***
**I67**		**0.0578**	***<0.0001***
**I69**		0.1069	***<0.0001***

* Only selected ICD-10 categories were tested due sample size limitations. Bold values highlight categories that had a significantly different ratio of diagnosis compared to the <25 years old reference category at 10% significance level and bold values in italics highlight ones significant at the 5% level; only diagnoses with at least 10 patients in the reference category were tested.

## Data Availability

Dataset available on request from the authors.
